# Life-long reduction in myomiR expression does not adversely affect skeletal muscle morphology

**DOI:** 10.1038/s41598-019-41476-8

**Published:** 2019-04-02

**Authors:** Ivan J. Vechetti, Yuan Wen, Thomas Chaillou, Kevin A. Murach, Alexander P. Alimov, Vandre C. Figueiredo, Maeli Dal-Pai-Silva, John J. McCarthy

**Affiliations:** 1 Department of Physiology, College of Medicine, University of Kentucky, Kentucky, USA; 2 Department of Rehabilitation Sciences, College of Health Sciences, Kentucky, USA; 3 Center for Muscle Biology University of Kentucky, Lexington, Kentucky, USA; 4 Department of Morphology, São Paulo State University, Institute of Biosciences, Botucatu, Brazil; 5 Örebro University, School of Health Sciences, Örebro, Sweden

## Abstract

We generated an inducible, skeletal muscle-specific *Dicer* knockout mouse to deplete microRNAs in adult skeletal muscle. Following tamoxifen treatment, *Dicer* mRNA expression was significantly decreased by 87%. Wild-type (WT) and *Dicer* knockout (KO) mice were subjected to either synergist ablation or hind limb suspension for two weeks. There was no difference in muscle weight with hypertrophy or atrophy between WT and KO groups; however, even with the significant loss of *Dicer* expression, myomiR (miR-1, -133a and -206) expression was only reduced by 38% on average. We next aged WT and KO mice for ~22 months following *Dicer* inactivation to determine if myomiR expression would be further reduced over a prolonged timeframe and assess the effects of myomiR depletion on skeletal muscle phenotype. Skeletal muscle *Dicer* mRNA expression remained significantly decreased by 80% in old KO mice and sequencing of cloned *Dicer* mRNA revealed the complete absence of the floxed exons in KO skeletal muscle. Despite a further reduction of myomiR expression to ~50% of WT, no change was observed in muscle morphology between WT and KO groups. These results indicate the life-long reduction in myomiR levels did not adversely affect skeletal muscle phenotype and suggest the possibility that microRNA expression is uniquely regulated in skeletal muscle.

## Introduction

MicroRNAs (miRNA) constitute a group of small non-coding RNAs that function through a post-transcriptional mechanism to control gene expression. Initially described as “short temporal RNA” in nematodes, miRNAs were more broadly recognized when *let-7* was found to be phylogenetically conserved across a range of species which subsequently resulted in the discovery of additional miRNAs in human, worm and fly^1–5^. Since that time, miRNAs have been identified in almost 300 species with over 38,000 mature miRNA sequences annotated in the miRBase database with over 2500 in humans alone^6^. The majority of human genes that encode a protein have been reported to contain conserved miRNA binding sites within their respective 3′-UTR, a finding that supports the growing recognition that miRNAs have an important role in many different cellular and developmental processes^7^.

As more and more miRNAs were identified, it became clear that some miRNAs were expressed in a tissue-specific manner and not ubiquitous as observed with *let-7*. One of the first examples of a miRNA that showed tissue-specific expression was miR-1 which was shown to be expressed only in cardiac tissue^3,5^. Lagos-Quintana and coworkers confirmed the discovery of tissue-specific miRNAs when they showed that miR-124a, miR-122 and miR-1 were only expressed in brain, liver and striated muscle, respectively^8^. Sempere and colleagues performed one of the first microarray analysis of miRNA expression across different tissues, identifying miR-1, miR-133a and miR-206 as muscle-specific; these miRNAs were later designated as myomiRs^9,10^. The myomiR family of miRNAs now includes miR-208a, miR-208b, miR-499 and, most recently, miR-486^11,12^. While the majority of myomiR members are expressed in skeletal and cardiac muscle, miR-206 is expressed exclusively in skeletal muscle whereas miR-208a is restricted to cardiac muscle^13^.

We published the first study showing that myomiR expression was significantly decreased during skeletal muscle hypertrophy and more recently showed by qPCR that miR-1 expression was down-regulated by ~60% following three days of mechanical overload^13,14^. We also provided some of the first evidence showing that expression of myomiR-499, a component of the myomiR network as described by the Olson laboratory, was decreased during muscle atrophy and was likely involved in the down-regulation of β/slow-myosin heavy chain gene expression through the upregulation of the myomiR-499 target gene Sox6^15^. These findings, together with other work in the field, suggest that myomiRs along with other miRNAs, have a role in regulating skeletal muscle mass. To test this idea, we generate a skeletal muscle-specific *Dicer* knockout mouse to deplete myomiRs levels in adult skeletal muscle.

Despite an almost 90% reduction in *Dicer* mRNA expression, average myomiR expression was only decreased by approximately 38% a month after *Dicer* inactivation with no effect on skeletal muscle hypertrophy or atrophy. Aging for an additional 22 months following *Dicer* inactivation resulted in a further reduction of myomiR levels to ~50% but, somewhat surprisingly, had no effect on skeletal muscle phenotype. These unexpected results indicate the life-long reduction in miRNA levels does not adversely affect skeletal muscle phenotype and, more importantly, suggest the intriguing possibility that miRNA expression is uniquely regulated in skeletal muscle.

## Materials and Methods

### Animal model

All animal procedures were conducted in accordance with institutional guidelines for the care and use of laboratory animals as approved by the Institutional Animal Care and Use Committee of the University of Kentucky. Mice were housed in a temperature- and humidity-controlled room and maintained on a 14:10-hr light-dark cycle with food and water *ad libitum*. To inactivate *Dicer* specifically in adult skeletal muscle, we crossed the skeletal muscle-specific inducible *Cre* mouse (HSA-MCM)^16^ with the floxed *Dicer* (*Dicer*^*fl/fl*^) mouse as described by Bernstein and coworkers to generate the HSA-MCM; *Dicer*^*fl/fl*^ mouse, designated HSA-Dicer. Following tamoxifen-induced Cre-mediated recombination, exons 23 and 24 were deleted from the *Dicer* gene in the *Dicer*^*fl/fl*^ mouse; exons 23 and 24 encode the RNAse III domain which is absolutely required for generation of mature miRNAs^17^.

### Skeletal muscle-specific inactivation of *Dicer*

Adult (~4 months of age) female and male HSA-Dicer mice were administered either vehicle (15% ethanol in sunflower seed oil) or tamoxifen (2 mg/day) by intraperitoneal injection for five consecutive days. Vehicle-treated mice were designated as wild-type (WT, female, n = 6; male, n = 3) and tamoxifen-treated mice designated as knockout (KO, female, n = 4; male n = 4). After one or 22 months (26 months old), WT and KO mice were humanely euthanized via carbon dioxide asphyxiation followed by cervical dislocation with hind limb musculature carefully excised, weighed and either prepared for immunohistochemistry or quick frozen in liquid nitrogen for downstream analysis of gene expression.

### Hind limb suspension and synergist ablation

Wild-type and Dicer KO mice were subjected to either synergist ablation or hind limb suspension to determine the effect of Dicer inactivation on muscle hypertrophy and atrophy, respectively. Synergist ablation and hind limb suspension were performed as previously described by us with hind limb musculature collected after 14 days for both procedures and prepared for either immunohistochemistry or quick frozen in liquid nitrogen^18,19^.

### Genotyping and recombination

Genomic DNA was isolated from 20 mg of tissue (liver, heart and gastrocnemius) using the DNeasy Blood & Tissue Kit (Qiagen) according to manufacturer’s protocol and screened by PCR for the presence of the *Dicer*^*fl/fl*^ allele (767 nt) or recombination (429 nt) as previously described by Berstein and colleagues using the following primers: 23F, 5′- ATTGTTACAGCGCTTAGAATTCC-3′; 458F, 5′-TCGGAATAGGAACTTCGT TTAAAC-3′ and 460R 5′-GTACGTCTACAATTGTCTATG-3′^20^.

### Immunohistochemistry

Hind limb muscles (plantaris, Pl; soleus, Sol; gastrocnemius, Gastroc) were mounted at resting length, covered in OCT compound (Tissue Tek, Sakura, Torrance, CA), frozen in liquid nitrogen–cooled isopentane and stored at −80 °C until sectioning. Immunohistochemistry protocols were based on those from Fry and colleagues^21^. For fiber-type staining, unfixed sections (7 µm) were incubated (90 min at RT) in antibodies to myosin heavy chain (MyHC) types 1, 2a and 2b (1:100 - BA.D5, SC.71 and BF.F3, respectively, Developmental Studies Hybridoma Bank) in addition to laminin (1:100, L9393, Sigma). Fluorescence-conjugated secondary antibodies to different mouse immunoglobulin subtypes (Gt anti-Ms IgG2b AF647, 1:250, Gt anti-Ms IgG1 AF488 1:500, Gt anti-Ms IgM AF555, 1:250 and AMCA Gt anti-Rb IgG (H + L) 1:150) were applied (1 hr at RT) to visualize MyHC expression and laminin. MyHC type 2x expression was inferred from unstained fibers. Sections were post-fixed in absolute methanol before mounting.

For Pax7 and laminin staining of plantaris muscle sections were fixed in 4% PFA, blocked in 1% Tyramide Signal Amplification kit (#T20935, Invitrogen, Carlsbad, CA) and incubated with anti-laminin antibody (1:100, L9393, Sigma) overnight at 4 °C. Following the overnight incubation, muscle sections were subject to epitope retrieval using sodium citrate (10 mM, pH 6.5) at 92 °C for 11 min. Endogenous peroxidase activity was blocked with 3% hydrogen peroxide in PBS followed by an additional blocking step with Mouse-on-Mouse Blocking Reagent (Vector Laboratories, Burlingame, CA). Incubation with anti-Pax7 antibody (1:100, Developmental Studies Hybridoma Bank, Iowa City, IA) was followed by incubation with the biotin-conjugated secondary antibody and streptavidin-HRP included within the Tyramide Signal Amplification kit (#T20935, Invitrogen, Carlsbad, CA). Sections were mounted with Vectashield fluorescent mounting media with DAPI.

For dystrophin staining, plantaris muscle sections were rehydrated with PBS and blocked in Mouse-on-Mouse Blocking Reagent. After washing, incubation with anti-dystrophin antibody (1:50, vector # VP D505) overnight (4 °C) was followed by incubation for 75 min with goat anti-mouse biotinylated secondary antibody (1:1000 in 1% TSA blocking, Jackson Immuno Research # 115-065-205). Sections were washed again, incubated 30 min in SA-FITC (1:150, Vector # SA-5001), and post-fixed in 4% paraformaldehyde before mounting using Vectashield fluorescent mounting media with DAPI. For N-acetyl-d-glucosamine staining, muscle sections were fixed with 4% PFA and then incubated with Texas red directly conjugated wheat germ agglutinin (WGA).

### Immunohistochemistry quantification

All cross-sections images were acquired with an upright microscope (AxioImager M1; Zeiss, Göttingen, Germany) at 20x magnifications. To capture the entire Pl, Sol and Gastroc muscle cross-section, 15–20, 9–12 and 110–120 images, respectively, were obtained. MyoVision analysis was used to quantify fiber-type composition, fiber-type specific cross-sectional area, and myonuclear number as described by us^22^. Fibers were classified as type 2A (green), type 2B (red) and type I (pink). Fibers that were counted as negative under all three channels were classified as type 2X. For the plantaris and soleus muscles, 400–800 fibers were counted per cross-section per animal whereas 2000–5000 fibers per cross-section were counted for each gastrocnemius muscle per. Satellite cell abundance was determined using the AxioVision Rel software (v4.8) with Pax7+/DAPI+ loci found within the laminin border scored as a satellite cell. Wheat germ agglutinin (WGA) staining was used to quantified the level of muscle fibrosis and employed a MATLAB script to calculate the total WGA area as previously described^23,24^.

### Single fiber analysis

Single fibers were isolated as previously described by Wada and coworkers^25^. Briefly, after careful removal of the gastrocnemius and soleus muscles, the plantaris muscle was left on the tibia and fixed *in situ* at resting length in 4% paraformaldehyde for 48 hr. Fixed muscle was divided into several bundles by pulling the tendon with tweezers and non-muscle tissue was removed. The muscle bundles were incubated in 40% NaOH for 3 hr at room temperature and then shaken to separate the bundle into single myofibers. Single myofibers were washed with PBS before being stained with DAPI for nuclear visualization. Suspended fibers were dispersed on a slide and mounted with Vectashield fluorescent mounting media. Images were captured using 20X magnification Z-stacks on an Axioimager MI upright microscope and all fiber and nuclear measurements were made using AxioVision Rel software (version 4.8). Twenty fibers from each animal were measured for fiber width (µm) and subsequently the nuclei from each fiber were counted to determine the number of myonuclei per defined fiber segment as described by Jackson and colleagues^18^.

### Gene expression

Total RNA was isolated from Gastroc muscle previously frozen in liquid nitrogen. Samples were homogenized in a tissue homogenizer (Bullet Blender, Next Advance Inc., Averill Park, NY) using TRIzol, according to the manufacturer’s instructions. Following isolation, RNA samples were treated with TURBO DNase (Ambion, Life Technologies) and cDNA was synthesized from 1 μg of total RNA using the SuperScript® VILO™ (ThermoFisher Scientific, Waltham, MA) according to the manufacturer’s instructions. Dicer (Mm00521730), Pri-miR-1A (Mm03306163) and Pri-miR-1B (Mm03308741) gene expression were analyzed using TaqMan gene expression assay (ThermoFisher Scientific, Waltham, MA), a 20-fold dilution of cDNA into a 20 µl final reaction.

### MiRNA expression

Reverse transcription (RT) reactions for miR-1, -133a, -206, -16, let-7b, let-7c and U6 small nuclear RNA (Rnu6) were performed with 10 ng of total RNA using Taqman MicroRNA Reverse Transcription Kit (ThermoFisher Scientific, Waltham, MA) according to the manufacturer’s directions. qPCR was carried out with Taqman Gene expression Master Mix (2x) (ThermoFisher Scientific, Waltham, MA), TaqMan gene expression assay (miR-1, #002222; miR-133a, #002246; miR-206, #000510; miR-16, #000391; Let-7b, 0000378; Let-7c, #000379; Rnu6, #001973) using cDNA in a 20 µL reaction volume.

### Quantification of mRNA and miRNA expression

qPCR reactions were performed in the ABI 7500 qPCR system as described by the manufacturer. qPCR efficiency was calculated by linear regression during the exponential phase using LinRegPCR software v11.1^26^. The comparison of mRNAs or miRNAs expression between WT and KO was determined following normalization with *Rpl38* (Mm03015864) or *Rnu6*, respectively. Relative quantification of mRNA and miRNA expression were assessed by using REST software 2009 v2.0.13^27^.

### Exon analysis

For exon deletion analysis, diluted cDNA was amplified by PCR with GoTaq® G2 Hot Start Green Master Mix (Promega) using the following primers: exon 21 forward, 5′-TGGGAACGCTAACACATCTACCTCAGA-3′ and exon 26 reverse 5′-CTGGTTCCATCTCGAGCAATTCTCTCACTG-3′. PCR products were gel purified (Zymoclean Gel DNA recovery Kit, Zymo Research) and then sequenced (Eurofin Genomics, Louisville, KY).

### Northern blot

To determine miR-1 expression, Northern blot analysis was performed using total RNA isolated from Gastroc muscles as described above. Denatured RNA (95 °C for 5 min in gel loading buffer) was separated on 15% urea–polyacrylamide gel (National Diagnostics) in a Mini-Protean vertical electrophoresis cell (Bio-Rad). To confirm equal loading prior to transfer, gels were stained with ethidium bromide (4 µg/ml) to determine tRNA abundance. Samples were transferred to positively charged, nylon membranes, using a Trans-Blot Semi-Dry Transfer Cell (Bio-Rad) for 75 min at 15 V in a cold room. Saturated 3MM Whatman chromatography paper in 1-ethyl-3-(3-dimethylaminopropyl) carbodiimide (EDC, Sigma) reagent was used to cross-link the RNA to the membrane for 2 hr 60 °C. After thoroughly rinsing the membrane with distilled water, ULTRAhyb hybridization buffer (Ambion) was used to pre-hybridize the membrane at 37 °C for at least 30 min. Following this step, the membrane was hybridized in the same solution using 0.5 nM of DIG-labeled LNA probe (5′-ATACATACTTCTTTACATTCCA-3′) following the manufacturer’s instructions (DIG Oligonucleotide Tailing Kit – 2^nd^ Generation, Roche) overnight at 37 °C. After hybridization the membrane was washed at low stringency in 2xSSC with 0.1% SDS at 37 °C twice for 15 min, at high stringency in 0.1 SSC with 0.1% SDS at 37 °C twice for five minutes, followed by 10 min at washing buffer 1xSSC at 37 °C. The membrane was then incubated in blocking buffer (DIG wash and block buffer set, Roche) for 3 hr at room temperature followed by incubation for 30 min with DIG antibody (1:15000) in blocking buffer. The membrane was then washed in DIG washing buffer four times for 15 min. Probe detection was performed using the DIG luminescent detection kit (CSPD, Roche) according to manufacturer’s protocol.

### Statistical analysis

All data was expressed as the mean ± SEM. Data were analyzed with GraphPad software (GraphPad Software, Inc., La Jolla, CA) using a two-tailed t-test for unpaired samples (aging experiments) or via a two-factor ANOVA (hypertrophy and atrophy experiments). If a significant interaction was detected, an appropriate post hoc analysis was employed to determine the source of the significance. Statistical significance was accepted at *p* ≤ 0.05.

## Results

**Figure 1:**
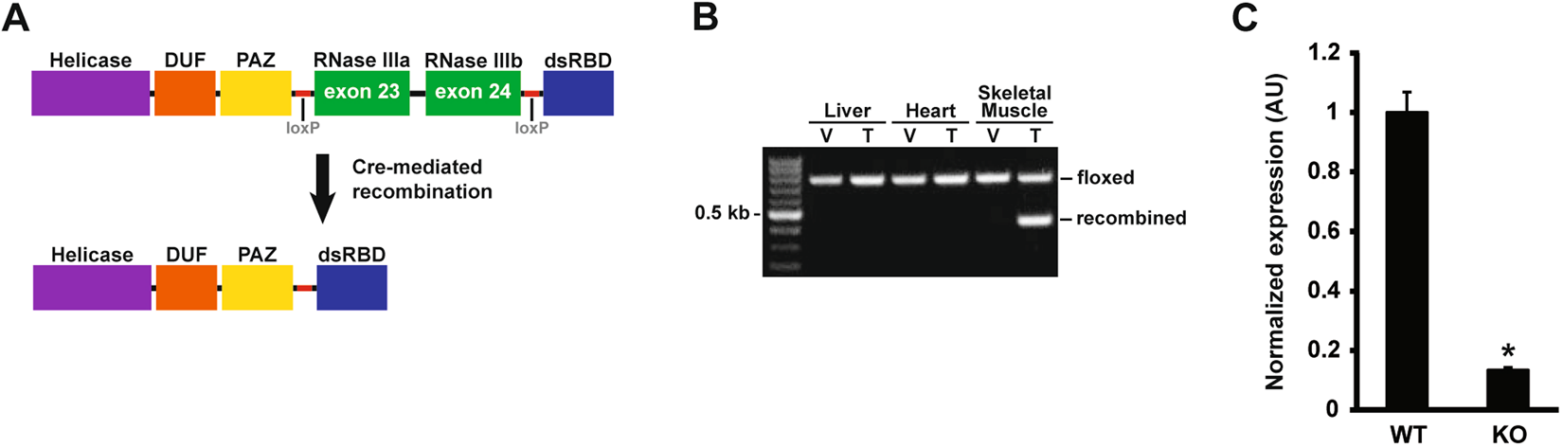
Skeletal muscle-specific Dicer knockout. (**A**) Scheme showing the major domains of the Dicer protein with loxP sites flanking exons 23 and 24 which encode RNase IIa and IIb domains, respectively; the RNAse II domain is absolutely required for generation of mature miRNAs. Following tamoxifen administration, Cre-mediated recombination causes excision of exons 23 and 24, thereby promoting *Dicer* inactivation. (**B**) PCR analysis using genomic DNA isolated from liver, heart or skeletal muscle shows Cre-mediated recombination (429 bp band) only occurs in skeletal muscle following tamoxifen administration. (**C**) *Dicer* mRNA expression was significantly lower (~90%) in skeletal muscle of tamoxifen-treated mice (knockout, KO) compared to vehicle-treated (wild-type, WT) mice. Values are presented as the mean ± SEM (n = 4–6) with expression normalized to WT. Asterisk denotes significant difference between WT and KO groups (*p* ≤ 0.05).

To determine the necessity of miRNAs in regulating skeletal muscle mass, we sought to deplete miRNAs by inactivating *Dicer* specifically in adult skeletal muscle. The myofiber-specific, inducible *Cre* mouse strain (HSA-MCM) was crossed to a floxed *Dicer* (*Dicer*^*f/f/*^) strain to generate HSA-MCM; *Dicer*^*f/f*^ mice, designated HSA-Dicer. Adult (at least 16 weeks of age) HSA-Dicer mice were treated with either vehicle (15% in sunflower seed oil) or tamoxifen (2 mg) and then recombination assessed using genomic DNA isolated from liver, heart or skeletal muscle. As shown in Fig. 1, Cre-mediated deletion of floxed exon 23 and 24 only occurred upon tamoxifen treatment and only in skeletal muscle. Quantitative PCR for the floxed exons showed that Cre-mediated recombination was highly effective with full-length *Dicer* expression significantly lower by 87% in the KO group compared to the WT group (see Fig. 1C).

**Figure 2:**
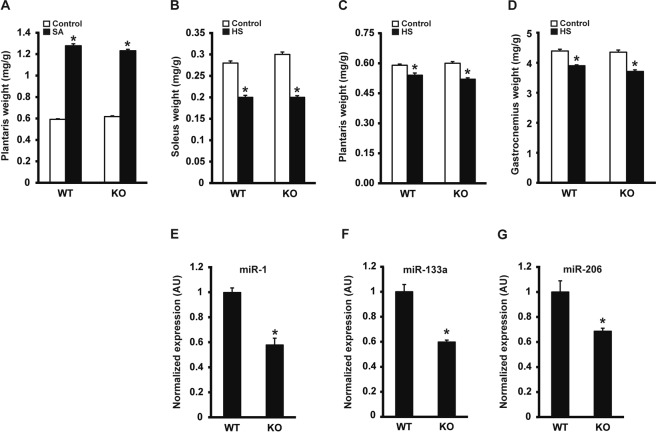
Skeletal muscle hypertrophy or atrophy are not affected in muscle-specific Dicer knockout. Following *Dicer* inactivation, mice were subjected to either synergist ablation (SA), to induce hypertrophy of the plantaris muscle or, hind limb suspension (HS), to induce muscle atrophy of hind limb musculature. (**A**) Following 14 days of SA, there was a significant increase in plantaris normalized muscle weight (mg/g) in both WT and KO groups compared to respective sham control but no difference between SA groups. (**B–D**) Following 14 days of HS, normalized soleus, plantaris and gastrocnemius muscle weight was significantly decreased in both WT and KO groups compared to respective ambulatory control with no difference between HS groups. (**E–G**) MyomiR (miR-1, -133a and -206) expression was significantly lower in KO compared to WT. Values are presented as the mean ± SEM (n = 4–6) with expression normalized to WT. Asterisk denotes significant difference between WT and KO groups (*p* ≤ 0.05).

We next determined the effect of *Dicer* inactivation on skeletal muscle hypertrophy and atrophy. Following two weeks of synergist ablation (SA), plantaris muscle weight was significantly increased by ~2-fold with no difference between WT and KO groups (see Fig. 2A). Conversely, following two weeks of hind limb suspension, the muscle weight of the soleus, plantaris and gastrocnemius muscles were significantly decreased approximately 30%, 11% and 13%, respectively, with no significant difference between WT and KO groups (Fig. 2B–D, respectively). Despite the almost 90% knockdown in *Dicer* mRNA, expression of the canonical myomiRs, miR-1, -133a and -206, were only decreased by 42%, 31% and 40%, respectively, following four weeks of inactivation (see Fig. 2E-G, respectively).

**Figure 3:**
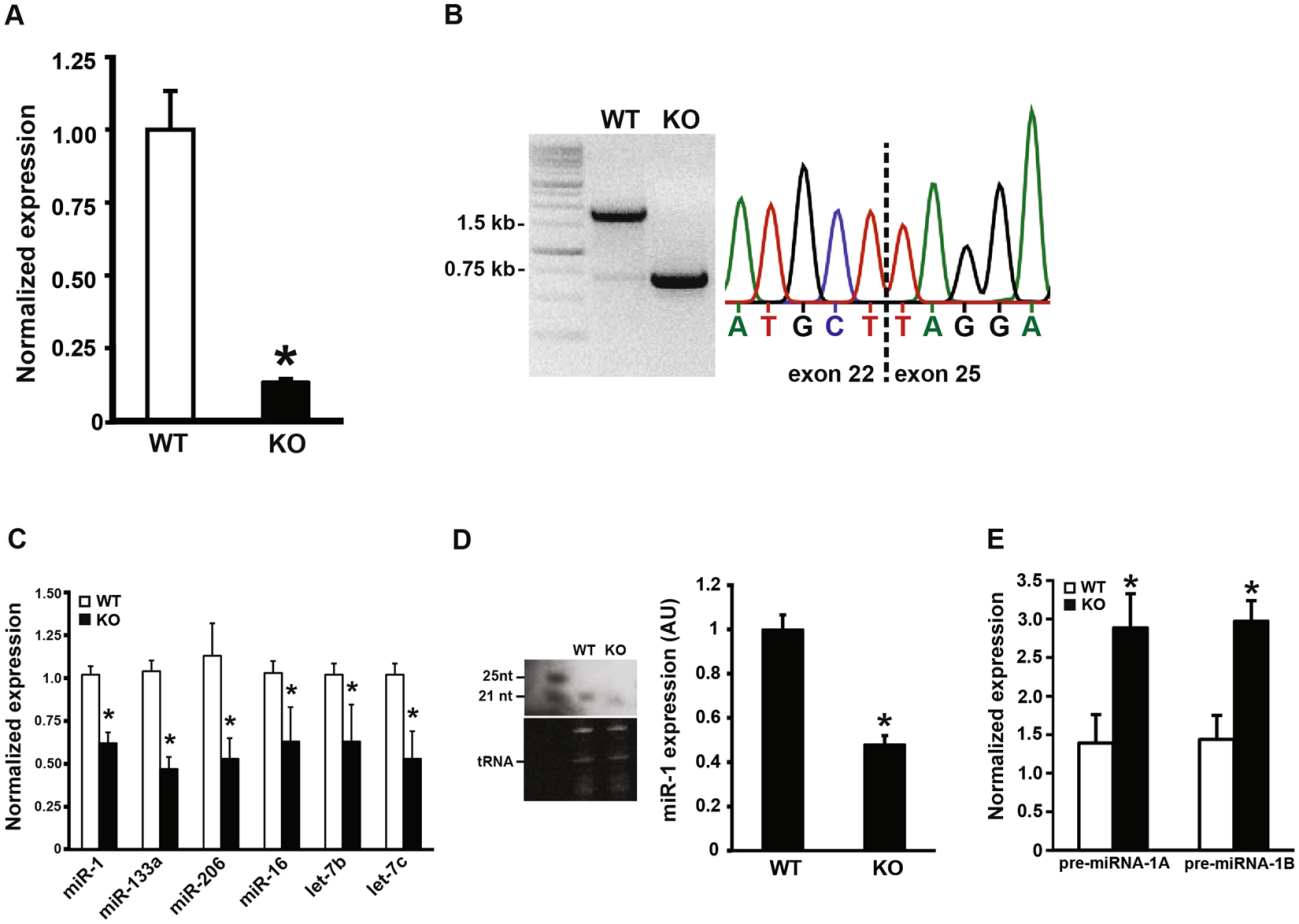
Aging of skeletal muscle-specific *Dicer* knockout mouse. Mice were treated with either vehicle (wild-type, WT) or tamoxifen (knockout, KO) at four months of age and then allowed to age for an additional 22 months. (**A**) *Dicer* expression was significantly decreased by 80% in skeletal muscle of KO mice compared to WT mice at 26 months of age. (**B**) Sequencing of a cloned fragment (exon 22–25) of *Dicer* mRNA isolated from skeletal muscle revealed deletion of floxed exon 23 and 24 in KO sample (0.75 kb fragment) whereas WT sample contained full-length fragment of ~1.5 kb. Trace from sequencing showed joining of exon 22 and 25 resulting from the deletion of exons 23 and 24 in KO sample. (**C**) qPCR analysis showed myomiR (miR-1, -133a and -206) and non-myomiR (miR-16, let-7a and let-7b) expression remained significantly lower with aging in skeletal muscle of KO mice compared to WT mice. (**D**) Northern blot analysis showed miR-1 expression was significantly lower by ~50% in skeletal muscle of KO mice compared to WT mice, consistent with qPCR results presented in panel C. (**E**) qPCR analysis showed a two-fold increase in both miR-1 primary miRNA (pri-miR-1A or -1B) transcript levels in skeletal muscle of KO mice compared to WT mice indicating an inability to effectively process the pri-miRNA transcript. Values are presented as the mean ± SEM (n = 8–9) with expression normalized to WT. Asterisk denotes significant difference between WT and KO groups (*p* ≤ 0.05).

The relatively modest decrease in myomiR expression following *Dicer* inactivation suggested that myomiRs, and likely miRNAs in general, might be very stable in adult skeletal muscle, requiring a longer period of time to achieve the 80–90% depletion of miRNA levels observed in skeletal muscle and other tissues following *Dicer* inactivation^20,28–35^. To test this idea, we aged WT and KO mice for an additional ~18 months and then assessed skeletal muscle phenotype. As shown in Fig. 3A, *Dicer* mRNA expression remained significantly lower by 80% in 22-month-old KO muscle compared to WT. To confirm deletion of *Dicer* exon 23 and 24, we cloned the portion of the *Dicer* mRNA containing exons 22–25 from a 22-month-old skeletal muscle cDNA library generated from either WT or KO mice. As shown in Fig. 3B, no full-length *Dicer* mRNA was detected in KO muscle and sequencing of the clone *Dicer* mRNA KO fragment reveal deletion of exons 23 and 24.

We next performed qPCR to determine if myomiR expression was further reduced with aging in the *Dicer* KO. As shown in Fig. 3C, expression of miR-1, -133a and -206 were significantly lower by 38%, 53% and 47%, respectively, in KO skeletal muscle compared to WT; however, the average decrease in myomiR expression was modest with aging as it went from 38% in young KO skeletal muscle to 46% in aged KO skeletal muscle. We also observed a similar significant decrease of ~40% in the expression of non-myomiRs (miR-16, let-7b and let-7c) (see Fig. 3C). In agreement with the qPCR results, Northern blot analysis showed a marked reduction in miR-1 expression in the KO compared to WT (see Fig. 3D). Expression of primary miRNA-1A and -1B (pri-miRNA-1A/B) was significantly higher by 2.1-fold in *Dicer* KO skeletal muscle compared to WT, a finding consistent with the loss of Dicer activity (see Fig. 3E).

**Figure 4:**
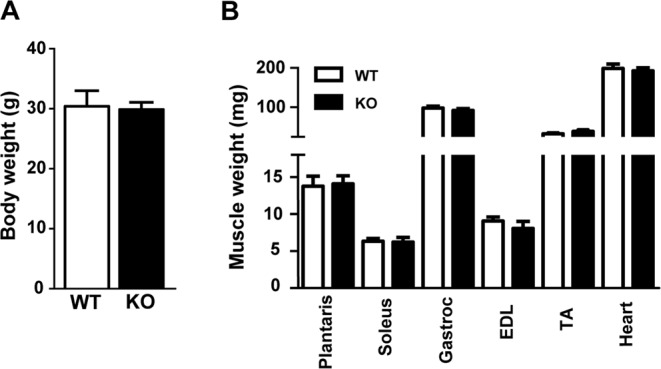
Body and muscle weight of aged skeletal muscle-specific Dicer knockout mouse. (**A**) No difference was observed in the body weight (mg) of 26 month old vehicle-treated (wild-type, WT) and tamoxifen-treated (knockout, KO) mice which were treated at four months of age. (**B**) No difference was observed in the weight of hind limb muscles (plantaris, soleus, gastrocnemius, EDL, TA) and heart of KO and WT mice. Values are presented as the mean ± SEM (n = 8–9).

**Figure 5:**
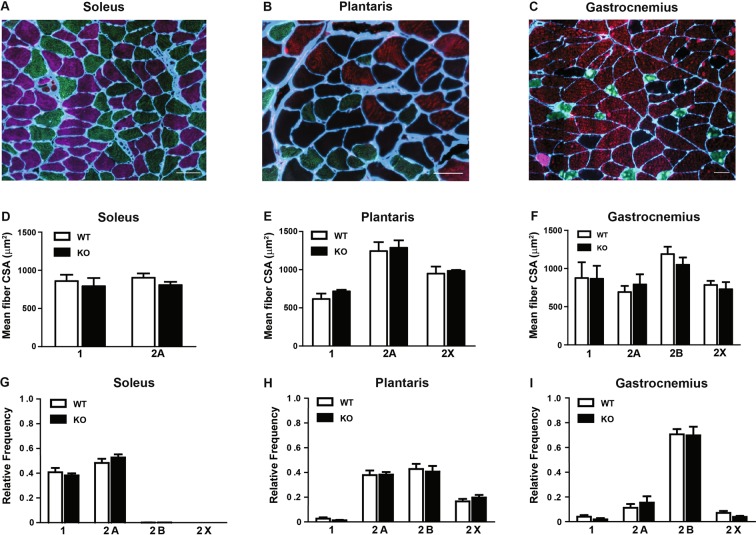
Muscle fiber size and fiber-type composition of aged skeletal muscle-specific Dicer knockout mouse. No difference was observed in fiber-type–specific cross-sectional area (CSA) of the soleus (type 1 and 2A), plantaris (type 2A, 2B and 2X) and gastrocnemius (type 1, 2A, 2B and 2X) muscles of vehicle-treated (wild-type, WT) and tamoxifen-treated (knockout, KO) mice that were treated at four months of age and then aged until 26 months of age. Representative cross-section image from the soleus (**A**), plantaris (**B**) and gastrocnemius (**C**) muscles are shown with myosin heavy chain type 1 (pink), 2A (green), 2B (red) and 2X (unstained) fibers with laminin (blue) demarcating the fiber border. Scale bar represents 50 µm. (**D–F**) Quantification of fiber-type-specific CSA for the soleus **(D)**, plantaris **(E)** and gastrocnemius **(F)** muscles of WT and KO mice. (**G–H**) No difference was observed in the fiber-type composition of soleus **(G)**, plantaris **(H)** and gastrocnemius **(I)** muscles from WT and KO groups. Values are presented as the mean ± SEM (n = 8–9).

**Figure 6:**
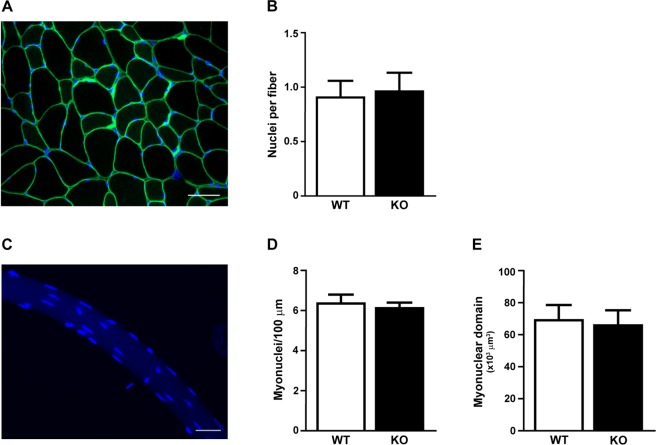
Myonuclear abundance of aged skeletal muscle-specific Dicer knockout mouse. (**A**) Representative image of plantaris muscle cross section stained with dystrophin (green) and DAPI (blue) to visualize the myofiber membrane and nuclei, respectively. Nuclei with their geometric mean within the dystrophin border were counted as myonuclei; scale bar, 50 μm. (**B**) No difference in myonuclear abundance of vehicle-treated (wild-type, WT) and tamoxifen-treated (knockout, KO). (**C**) Representative image of a single plantaris myofiber with myonuclei visualized with DAPI; scale bar, 50 μm. (**D**) DAPI + myonuclei were counted in fixed single fibers isolated from WT and KO plantaris muscles and presented as myonuclei per fiber length (**E**) No difference in the calculated myonuclear domain size between WT and KO groups. Data is presented as mean ± SEM (n = 5–6).

**Figure 7:**
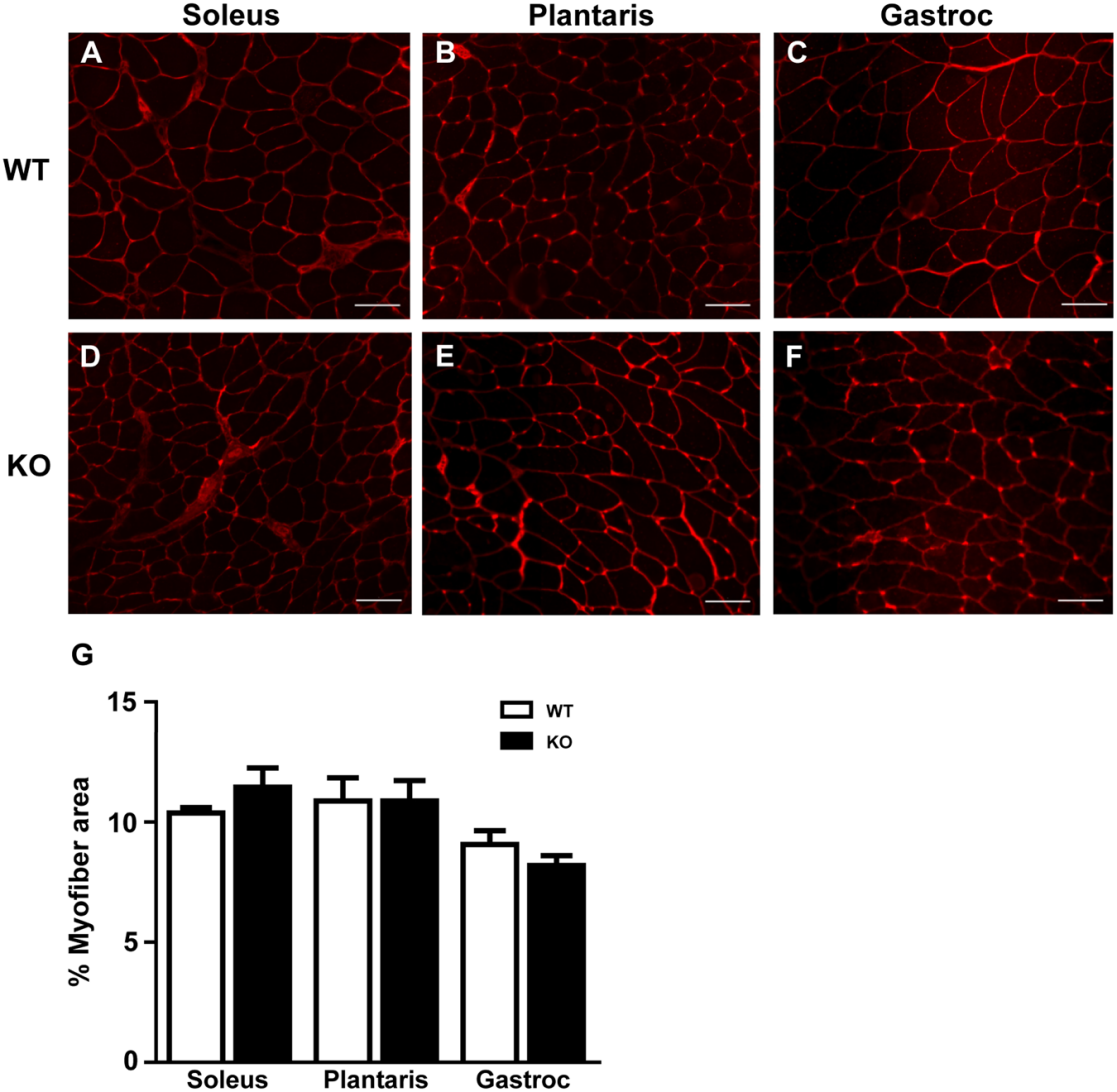
Extracellular matrix deposition of aged skeletal muscle-specific Dicer knockout mouse. (**A–F**) Representative image of WGA staining (red) of hind limb muscles from vehicle-treated (wild-type, WT) and tamoxifen-treated (knockout, KO) mice for soleus (**A,D**), plantaris (**B**,**E**) and gastrocnemius (gastroc) (**C**,**F**); scale bar, 50 μm. (**G**) Quantification of WGA staining in WT and KO groups presented as a percentage of the total cross-sectional area. Data are presented as the mean percent (%) area ± SEM (n = 8–9).

**Figure 8:**
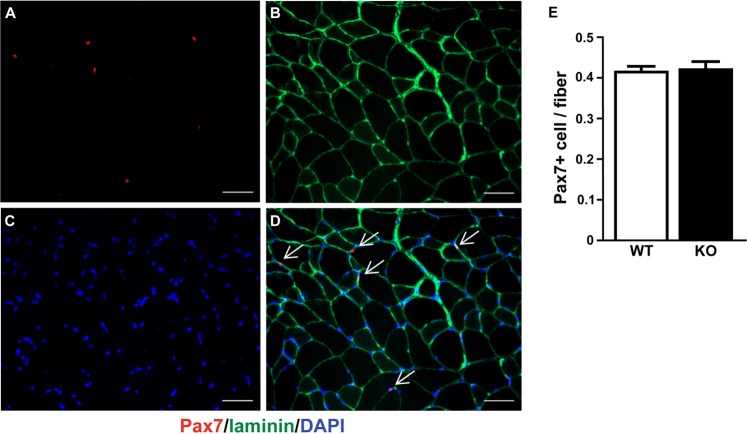
Satellite cells content after 22 months of Dicer inactivation. Pax7 (Pax7+) immunohistochemistry was used to detect satellite cells in plantaris muscle cross-section. (**A**) Representative image of Pax 7+ (red loci) co-stained with laminin (green boundary) and DAPI from vehicle-treated (wild-type, WT) plantaris muscle. Scale bar = 50 μm. (**B**) Quantification of satellite cell abundance in plantaris muscle from WT and tamoxifen-treated (knockout, KO) mice. Data are presented as the mean ± SEM (n = 8–9).

Although we were unable to deplete miRNAs to levels typically reported in other tissues following *Dicer* inactivation, we thought it would still be informative to determine the effect of a ~50% depletion of myomiRs on skeletal muscle phenotype. As shown in Fig. 4A-B, there was no difference in body weight or the weight of various skeletal muscles (plantaris, soleus, gastrocnemius, EDL or TA) or heart between WT and KO groups. In agreement with the muscle weight results, there was no difference in the fiber-type specific muscle fiber cross-sectional area of the soleus, plantaris or gastrocnemius muscles between WT and KO groups (see Fig. 5A–D). Moreover, as shown in Fig. 5E, there was no change in the fiber-type composition of the soleus, plantaris or gastrocnemius muscles of KO mice in comparison to WT mice; this analysis also demonstrated there was no difference in muscle fiber number for each of the different muscles under investigation. Myonuclear abundance, as assessed by cross-section or single fiber, was not different between WT and KO groups; accordingly, the calculated myonuclei/fiber length from the single fiber analysis was the same between WT and KO groups (see Fig. 6A–D). There also was no difference in the calculated myonuclear domain size between WT and KO groups (see Fig. 6E). As presented in Fig. 7, wheat germ agglutinin staining revealed no difference in the level of skeletal muscle fibrosis of the soleus, plantaris or gastrocnemius muscles between WT and KO groups. Finally, as shown in Fig. 8, Pax7 immunohistochemistry of plantaris muscle cross-sections showed no difference in satellite cell abundance normalized to fiber number between WT and KO groups.

## Discussion

The initial purpose of the present study was to test the hypothesis that miRNAs are necessary to regulate skeletal muscle mass in response to either a hypertrophic or atrophic stimulus. To test this hypothesis we generated an inducible, skeletal muscle-specific *Dicer* knockout mouse designated HSA-*Dicer*. We chose to use the myofiber-specific inducible Cre (HSA-MCM) mouse because it has been reported to promote effective recombination in both slow- and fast-twitch fibers with minimal expression in the heart^16^. Myofiber-specific expression of inducible Cre is driven by the human skeletal muscle α-actin promoter (HSA); the HSA promoter contains 2,000 bp of human skeletal α-actin 5′-flanking sequence plus the first exon and 149 bp of the first intron and shown by Muscat and Kedes to promote robust, skeletal muscle-specific expression^36^. Following tamoxifen treatment, Dicer mRNA expression was 87% lower compared to WT mice, thus indicating effective inactivation of *Dicer*. The inactivation of *Dicer* resulted in deletion of exons 23 and 24 which encode the RNase III domain of the DICER protein; the RNase III domain is absolutely necessary to cleave the precursor miRNA stem loop to generate the mature miRNA^37–41^.

We hypothesized that the depletion of miRNAs following *Dicer* inactivation would affect the ability of adult skeletal muscle to adapt to a change in use. Based on our previous work, we thought the depletion of miR-1 in particular would enhance skeletal muscle hypertrophy as well as protect against muscle atrophy^14,42^. This hypothesis is supported by evidence showing miR-1 targets pro-growth genes such as IGF-1 and cyclin D^43–45^. Despite the significant knockdown of *Dicer* mRNA expression, we observed no difference in the change in skeletal muscle mass between WT and KO in response to either a hypertrophic or atrophic stimulus. To confirm *Dicer* inactivation resulted in miRNA depletion, we assessed by qPCR myomiR expression in WT and KO skeletal muscle; however, despite robust *Dicer* inactivation, myomiR (miR-1, -133a and -206) expression was only lowered by ~40% in the KO compared to WT skeletal muscle. We also found that non-myomiR (miR-16, let-7a and Let-7b) expression was reduced by a similar magnitude indicating myofiber miRNA expression in general was depleted by approximately 40% following a lifetime of *Dicer* inactivation. This finding was completely unexpected given the previous success of this floxed *Dicer* strain to effectively deplete miRNAs in other tissues^20,29–35^. Thus, the relatively modest depletion of myomiRs in KO muscle more than likely accounts for there being no difference in the hypertrophic or atrophic response between WT and KO skeletal muscle.

Our results are in stark contrast to O’Rourke and colleagues who reported a decrease in skeletal muscle mass as the result of hypoplasia following skeletal muscle-specific *Dicer* inactivation during embryonic development (E9.75–E12.5)^28^. This dramatic phenotype was associated with a ~80–90% decrease in myomiR expression after two days of *Dicer* inactivation; moreover, the significant reduction in myomiR expression occurred despite there still being an estimated 30% of wild-type *Dicer* transcript present^28^. We, on the other hand, were able to knockdown *Dicer* expression by almost 90% but myomiR expression by only roughly 40%. Further, as O’Rourke and coworkers suggested, the incomplete knockdown of *Dicer* expression is the result of *Dicer* expression in non-muscle tissue found within skeletal muscle such as fibroblasts, endothelial and neural tissue. Although we do not have a definitive answer, we speculate the discrepancy in the effectiveness of myomiR depletion between the two studies is likely related to how the loss of *Dicer* differentially effects miRNA turnover in mitotic versus post-mitotic cells - i.e., embryonic vs. adult skeletal muscle^46^.

While the results of the hypertrophy and atrophy studies were inconclusive because of the relatively modest decrease in myomiR expression, they did suggest to us that myomiRs may be quite stable in adult skeletal muscle. As an initial effort to test this idea, we aged mice for 22 months following myofiber-specific *Dicer* inactivation and then measured myomiR expression as well as assessed skeletal muscle phenotype. Although *Dicer* expression remained significantly lower in aged skeletal muscle of KO compared to WT, myomiR expression only showed a further decrease to ~50% in aged KO skeletal muscle. Northern blot analysis confirmed that miR-1 expression was reduced by approximately 50% in the KO compared to WT in agreement with the qPCR results.

The finding that myomiR expression was only reduced on average 50% following life-long *Dicer* inactivation was perplexing given the reported effectiveness of *Dicer* inactivation to deplete miRNA levels in other tissues^20,29–35^. To confirm our qPCR results showing effective recombination following tamoxifen treatment, we cloned from WT or KO skeletal muscle cDNA libraries a fragment of the *Dicer* mRNA encompassing exons 22–25. Sequencing of the cloned fragment confirmed that we had in fact successfully deleted exons 23 and 24 in KO skeletal muscle. Moreover, as shown in Fig. 3B, we were unable to detect any full-length *Dicer* fragment in KO skeletal muscle, although we were able to detect a small amount of the recombined fragment in WT skeletal muscle. Together with our qPCR result, this finding provided compelling evidence that we had successfully induced highly effective recombination following tamoxifen treatment which resulted in deletion of exons 23 and 24, the exons encoding the all-important RNase III domain of the Dicer protein.

One limitation of the study was our inability to reliably detect Dicer protein by Western blot analysis using WT and KO whole-muscle lysates. We used both commercial (ab167444, sc393328 and ABIN2253355) and researcher-derived (clone 1414 and 1416) Dicer antibodies but were unable to produce consistent results using the same samples^29^. So, while we were able to effectively knockdown *Dicer* mRNA levels by 80–90%, it remains possible translation of the remaining *Dicer* mRNA is able to generate a sufficient amount of Dicer protein to maintain myomiR levels at 50%. While we fully acknowledge this possibility, we think, as suggested above, that most of the remaining full-length Dicer mRNA is coming from non-muscle tissue found within skeletal muscle.

An alternative explanation is the possibility that myomiRs may be very stable in adult skeletal muscle. In trying to explain our results, we came to realize that it might be telling us something unique about the regulation of miRNAs in adult skeletal muscle - that miRNAs in adult skeletal muscle may be very stable. To the best of our knowledge, no studies have measured miRNA half-life in skeletal muscle, either *in vitro* or *in vivo*; however, a number of studies have determined miRNA stability in other tissues, reporting half-lives between 5–9 days, though some miRNAs are much less stable with half-lives of less than 24 hr^46,47^. Interestingly, the longest miRNA half-life reported to date was for cardiac-specific miR-208 hinting at the possibility that miRNAs might have unusually long half-lives in skeletal muscle^48^.

An alternative possibility is that miRNA expression is being “rescued” following *Dicer* inactivation by exosome delivery of miRNAs and/or Dicer protein. Over the past few years, extracellular vesicles (EVs) have emerged as novel mediators of intercellular communication through the delivery of miRNAs^49,50^. Support for such a mechanism comes from a recent study by Crewe and coworkers showing adipocyte-specific knockout of caveolin 1 was rescued by the transfer of caveolin 1-containing EVs to adipocytes from neighboring endothelial cells *in vivo*^51^.

The finding that miRNAs are indispensable for skeletal muscle development^28^, suggest they may also have an important role in adult skeletal muscle plasticity and maintenance; however, despite a life-long 50% reduction in myomiR expression, we were unable to detect any phenotypic change in skeletal muscle such as myofiber size or fiber-type composition. These finding suggests that reduction of less than 50% are well-tolerated in skeletal muscle and have important implications for miRNA studies investigating the role of miRNAs in sarcopenia. While speculative at this time, the results of our study suggest the intriguing possibility that miRNAs are extremely stable in skeletal muscle or, more likely, are regulated by an alternative mechanism that is skeletal muscle-specific.
